# A Series of Anatomical Sketches and Diagrams, with Descriptions and References

**Published:** 1839-10

**Authors:** 


Art. XIV.—1. *A Series of Anatomical Sketches and Diagrams, with Descriptions and References.* By Thomas Wormald, Assistant-Surgeon and Demonstrator of Anatomy at St. Bartholomew's Hospital; and Andrew M. M'Whinnie, Teacher of Practical Anatomy at St. Bartholomew's Hospital. Part II. *London*, 1839.

2. *The Surgical Anatomy of the Groin, the Femoral and Popliteal Regions.* By Thomas Morton, formerly one of the House-Surgeons of University College Hospital. *Illustrated with Lithographic Plates and Wood-Engravings.—London*, 1839. Roy. 8vo, pp. 207.

We have already had occasion to notice the preceding parts of both of the above works in recent Numbers of our Journal, (Vol. VI. p. 202, and Vol. VIII. p. 244.) The present subjects are equally well treated by their respective authors. The "Sketches" are particularly well executed; and we cannot but again express our approbation of the style which Messrs. Wormald and M'Whinnie have selected for their illustrations: their outlines are accurately and elegantly executed, and, to our taste, are far more satisfactory than the most highly-finished engravings would be. Mr. Morton's *illustrations* are drawn with more freedom than those which adorned his former number on the Perineum; but they strike us as being almost too much finished: a more sketchy style and rather less paint would save trouble, whilst the desired result would be more effectively obtained. Much care has been bestowed by Mr. Morton in collecting information relating to herniæ, popliteal aneurism, dislocations of the thigh, &c., from the most approved authorities; and the production is altogether one which we can conscientiously recommend to the *working* student. We observe on the fly-leaf that Mr. Morton promises two more parts to complete his work;—whilst Messrs. Wormald and M'Whinnie intend extending their sketches to six numbers: each work will, when entire, comprise "views of those regions of the body most important to the student and surgeon,"—while Mr. Morton's will constitute a complete and elaborate treatise that cannot fail to be highly useful to surgeons in general. It speaks highly for his school, and still more highly for the writer, when a work of this kind is the production of one who is almost yet a student.

